# EMANCIPATORY GERONTOLOGY IN THE 21ST CENTURY—STRUCTURE, ACTION, AND IDEOLOGIES: A SYMPOSIUM HONORING CARROLL ESTES

**DOI:** 10.1093/geroni/igae098.0176

**Published:** 2024-12-31

**Authors:** Dale Dannefer, Chris Phillipson, Jessica Kelley

**Affiliations:** Chair; Co-Chair; Discussant

## Abstract

Emancipatory gerontology (Estes, 2020) envisions possibilities of moving toward a society where citizens of all ages experience dignity and access to essential resources. Such possibilities require transforming institutional configurations and dominant ideological narratives around class, race, gender, and age. Yet the longstanding resistance to such ideals has been further aggravated recently by the effects of neoliberal policies and by renewed forms of racist and other exclusionary ideologies, as well as ageism itself. The symposium presents critical work across a range of key gerontological domains. Papers explore the stratification of longevity and related issues, especially in urban settings, ideological and practical consequences of the intersection of age and race, and ongoing conceptual and ideological issues in life-course and gerontological theorizing. Maya Rockeymoore Cummings elaborates the fresh concept of rageism - the intersection of ageism and racism - and the ways in which various levers of power are used to undermine life chances of non-European racial and ethnic groups across the life course and generations. Jan Baars explores inequalities affecting ageing over the life course, arguing that long lives are increasingly a privilege for wealthier members of society. Chris Phillipson and Tine Buffel consider new social divisions emerging between and across age groups, with large cities driving distinctive forms of inequality associated with gentrification and class and race segregation. Dale Dannefer interrogates the continuing uncritical use of the concept of agency, exploring how discourses of life-course and gerontological scholarship may have ideological components that contributes to the legitimation of destructive social arrangements.

